# Domestic Double Burden of Malnutrition in Manisa, Turkey: A Cross-Sectional Study in Two Primary Care Health Centers

**DOI:** 10.7759/cureus.70793

**Published:** 2024-10-03

**Authors:** Ozge Longwill

**Affiliations:** 1 General Practice, Manisa Celal Bayar University, Manisa, TUR

**Keywords:** dbm, double burden of malnutrition, malnutrition risk, maternal obesity, pediatric malnutrition, pediatric obesity

## Abstract

Background

Child growth and development are profoundly influenced by postnatal nutrition. Despite global efforts, malnutrition and obesity remain pressing issues. In Turkey, child stunting and maternal obesity are significant concerns, with emerging cases of double burden of malnutrition (DBM), where households face both undernutrition and obesity.

Aims and objectives

This study aims to determine the prevalence of DBM at the household level in Manisa, Turkey, and identify associated factors. Objectives include assessing the nutritional status of children and mothers and exploring sociodemographic and lifestyle factors related to DBM.

Methods

A cross-sectional study was conducted with 385 women and their children (0-5 years) from two family health centers. Data were collected via interviews and anthropometric measurements. Descriptive statistics and logistic regression analyses were performed using Statistical Product and Service Solutions (SPSS, version 23.0; IBM SPSS Statistics for Windows, Armonk, NY) to evaluate DBM prevalence and associated factors.

Results

The study highlights that 63.2% of mothers are overweight or obese, with significant associations with lower education levels, inadequate health insurance, and financial instability. Among children, 7.8% were obese and 15.1% were stunted, with significant links to age, birth weight, and breastfeeding practices. The double DBM, where both maternal obesity and child malnutrition coexist, was present in 15.9% of households. Migration patterns, economic challenges, and price increases affected dietary choices and overall health. Notably, birth order and type of health insurance were significant predictors of the double DBM.

Conclusion

The study underscores the interplay between socioeconomic factors and nutritional status in both mothers and children. High rates of maternal obesity and child malnutrition, including stunting and obesity, are linked to education, income, and health insurance status. Addressing these socio-economic disparities and improving access to healthcare and nutrition is essential to mitigate the double DBM and enhance overall health outcomes.

## Introduction

Child growth and development is a dynamic process influenced by environmental conditions. Starting from the postnatal period, the environmental conditions to which a child is exposed can affect optimal growth. It is of utmost importance that nutrition and growth are closely monitored from the postnatal period onwards to investigate nutrition's influence on growth [1]. Commonly used indicators when evaluating nutritional status in children include height-for-age (HFA), weight-for-age (WFA), and weight-for-height (WFH) [2]. Over the past 20 years, significant progress has been made in enhancing maternal and child nutrition, with a notable reduction of one-third in the prevalence of stunted growth among children. However, the triple burden of malnutrition - stunting, wasting, and overweight - continues to threaten children's survival and overall well-being. World Health Organization (WHO) 2022 report showed that 6.8% of children under five years of age in the world are affected by wasting (WFH), 5.7% are overweight (WFA) and 22.3% are stunted [3]. The United Nations International Children's Emergency Fund (UNICEF) 2014 report found that 12.0% of children under the age of five in Turkey were stunted, 1.0% were underweight, and 2.0% were moderately to severely underweight (WFA) [4]. A 2018 report showed that 6.0% of children under five in Turkey were found to be stunted, with 1.5% experiencing severe stunting [5].

In addition to malnutrition, obesity has become a serious health problem worldwide [1]. The WHO defines obesity as "abnormal or excessive accumulation of fat in the body to the extent that it impairs health". According to WHO 2016 data, 40.0% of women over the age of 18 are overweight, and 15.0% are obese [6]. The 2019 Turkey Health Survey (THS) established that 24.8% of women were obese and 30.4% were overweight [7]. People in the poorest fifth of countries are more likely to be overweight or obese [8].

The increase in industrial food consumption and changing living conditions have led to a novel occurrence called the double burden of malnutrition (DBM), where both malnutrition and overweight/obesity coincide in the same population or individuals [9]. The DBM at the household level is when one person in the household is overweight/obese while another family member is underweight/stunted. Within a household, DBM can be found in four different forms: a child may be stunted and overweight at the same time, a mother may be overweight and one of her children younger than five years of age may be underweight or stunted, or a mother may be underweight and one of her children may be overweight [10].

In a 2007 study conducted in Mexico, maternal central obesity and child stunting were both present in 6.0% of mother-child pairs [11]. A cohort study in Kenya found that 43.0% of children of overweight mothers and 37.0% of children of obese mothers were found to be stunted [12]. A 1998 study conducted in the Cape peninsula of South Africa concluded that a subset of the population deprived of nutrition during childhood missed optimal nutrition and became severely overweight later in life [13]. In 2001, a study conducted in Southeast Asian and Pacific countries found that the DBM was a major issue. Then, in 2014, the situation worsened as they discovered stunting in more than 30.0% of children and overweight in more than 25.0% of women in the same household [14,15]. In 2014, a review of national data from 1998, 2002, and 2008 in Guatemala revealed that child stunting and maternal overweight coincided in 20.0% of households [16].

Currently, malnutrition, which causes 49.0% of under-5 mortality, is an important public health problem in underdeveloped and developing countries [17]. Malnourished children may suffer from many acute, chronic, and serious conditions such as cognitive developmental delay; frequent infections; poor performance at school; adult obesity; and an increased susceptibility to non-communicable diseases [18-22].

Among the risk factors for malnutrition, maternal age and education level, number of pregnancies, socioeconomic status of the family, desirability of pregnancy, birth weight, duration of breastfeeding, and maternal obesity are ranked highly [9,19]. The main causes of maternal obesity today are the more frequent consumption of processed foods with low nutritional value, which have become cheaper and more accessible, and the decrease in time allocated to physical activity. Over-processed foods lead to obesity in adults due to their high-calorie content, it has been predicted that ingesting these foods in the first 1,000 days of life will cause stunting in the future [8]. Food insecurity, chronic disease affliction, social and demographic constraints, increased urban migration, lack of physical activity, and increased food supply led to an increase in overweight mothers, at the same time contributing towards retarded growth and development in children [18-21]. This study aims to determine the DBM at the household level and associated factors among individuals attending Manisa Şehzadeler Family Health Center (FHC) No. 4 and Yunusemre Family Health Center (FHC) No. 12.

Despite extensive research on malnutrition and obesity, there remains a notable gap in understanding the simultaneous occurrence of both conditions within households, particularly in specific regional contexts such as Manisa, Turkey. Global and national studies have identified trends and statistics related to malnutrition and obesity separately, but there is limited insight into how these issues interact within the same households and how they impact different family members. This study seeks to fill this gap by examining the DBM at the household level, focusing on the interplay between undernutrition and overweight/obesity. The significance of this research lies in its potential to provide a nuanced understanding of the double burden phenomenon in a specific locality, which could inform targeted public health interventions and policies. By identifying the prevalence and determinants of DBM in Manisa, the study aims to contribute valuable data that can enhance strategies for addressing both malnutrition and obesity, ultimately improving health outcomes and guiding effective resource allocation in similar settings.

## Materials and methods

Study design

This study employed a cross-sectional design, with data collected through face-to-face questionnaire surveys and anthropometric measurements. The research was conducted in compliance with ethical standards, with approval obtained from the Ethical Committee of Manisa Celal Bayar University prior to the commencement of the study.

Targeted area

The targeted area of this research is Manisa Şehzadeler Family Health Center (FHC) No. 4 and Yunusemre Family Health Center (FHC) No. 12.

Eligibility criteria

It included women with children aged 0-5 years who attended the designated FHC in person.

Sample size

The sample size of this study is 385, including women with children aged 0-5 years who are eligible for inclusion criteria. We will calculate our sample size using standard online tools through the following formula $n = (z\alpha)^{2} \times ([p(1-p)/d^{2}])$.

Where:

n = estimated sample size.

zα at 5% level of significance = 1.96

d = level of precision and is estimated to be 0.05

p = Proportion of previous studies (0.5)

Actual sample size = (Primary sample size × design effect (estimated to be 1.5)

The expected response rate is estimated to be 80%.

z = 1.96, p = 0.5, d = 0.05\\ n = (1.96)^{2}\times([0.5\times(1-0.5)]/0.5^{2})\\ n = 0.9604 / 0.0025 = 384.16\\ n \approx 384\\

The sample size is equal to 384.

Study’s period

The study’s duration is between 3 and 28 October 2022.

Data collection

Data were collected using a 65-question questionnaire during face-to-face interviews. The questionnaire form was administered to women with children aged 0-5 years being seen at the FHCs who agreed in person by verbal consent to participate in the study. The weights and heights of the children and mothers were then measured using an electronic scale and tape measure at the FHCs. The questionnaire forms took approximately 15-20 minutes to complete and were accomplished by self-reporting in a single sitting. The number of people participating in the study was 258. The participation rate of the study was 67.2%.

Ethical considerations

All participants were informed of the confidentiality and anonymity of the study. No identifying information, such as names, was collected at any stage of the research. The surveys were designed to protect participants' privacy, and all data were stored securely to prevent unauthorized access.

Dependent variables

The dependent variables are HFA, WFA, and WFH of the child, maternal overweight-obesity, and the double burden of malnutrition; child's global developmental delay (GDD) and maternal overweight-obesity, child's weight-for-gestational age (WGA) and maternal overweight-obesity; and child's birth gestational age (BGA) and maternal overweight-obesity.

The HFA, WFA, and WFH z scores were calculated using the age, height, and weight values of each child. The calculation was made by subtracting the median value of the reference group of the same age and sex from the anthropometric measurement of the child and dividing it by the standard deviation of the reference group. National Center for Health Statistics (NCHS) and Centers for Disease Control and Prevention (CDC) standards recommended by WHO were used as the reference population. Children with z scores of -2 standard deviation and below were considered stunted, underweight, and wasted [23].

The double burden of malnutrition at the household level occurs when a mother is overweight or obese and her child is undernourished [24]. A mother is defined as overweight or obese if her BMI is 25 kg/m^2^ or more. A child is considered malnourished if he/she is stunted, underweight, or thin. The scores of the children and the body mass index (BMI) of the mothers were first considered individually and then paired to obtain two other variables representing the double burden of malnutrition. These variables are KM 7, a child’s persistent developmental delay (PDD).

Independent variables

Socio-demographic status and lifestyle, maternal and child characteristics, pregnancy and childcare, and healthy eating characteristics are independent variables.

Mother's age, child's age, marital status, age at first marriage, spouse's age, educational status, spouse's educational status, employment status, spouse's occupation, family type, income level, health insurance, migration status, region and year of migration, presence of chronic diseases in mother and child, knowing the family physician, permission required from the spouse when going out, permission required from the spouse when shopping are all sociodemographic status and lifestyle variables.

Maternal and child characteristics include birth weight, mode of delivery, number of children in the family, birth order of the child, and the mother's age at childbirth.

Pregnancy and child care characteristics include the desired pregnancy status, number of follow-ups during pregnancy, smoking during pregnancy, number of months of breastfeeding, time of first breastfeeding after birth, exclusive breastfeeding status for the first six months, time of starting complementary foods, vitamin D intake status of the child, iron supplementation status of the child, and number of diarrhea and upper respiratory tract infections in the last year.

Characteristics of healthy eating include the effect of the increase in food prices in the last year on household nutrition, the change in purchasing power in the last five years, accessibility to healthy foods, frequency of buying packaged foods at home, checking the expiration date and ingredients of foods, thinking that they have a healthy diet, physical activity status, and Mediterranean diet adherence scale.

The Mediterranean diet adherence scale is a questionnaire consisting of 14 questions, including the main type of fat/oil used in meals; the amount of olive oil consumed daily; fruit and vegetable portions; margarine-butter and red meat consumption; weekly consumption of wine, legumes, fish-seafood, nuts, seeds, tomato sauce with olive oil; and whether white meat is preferred more than red meat. Depending on the level of consumption, a score of 1 or 0 is obtained for each question, and the total score is calculated.

A total score of 7 and above indicates that the individual has acceptable compliance with the Mediterranean diet, and a score of 9 and above indicates that the individual has strict compliance with the Mediterranean diet. The questionnaire informs us whether the patient has Mediterranean-style eating habits [25].

For the definition of social class, the father's job was determined via Korkut Boratav's urban social class schema and reduced to two categories in the analysis: lower and upper social class [26].

Data management

Data management involved secure storage of completed questionnaires, with immediate coding for anonymity. A double-entry verification process minimized errors and was backed up regularly to prevent data loss. The dataset was organized into dependent and independent variables. Missing data were addressed ethically, either through imputation or exclusion. Access was restricted to authorized personnel, with confidentiality agreements and security measures in place to protect data integrity. An audit trail tracked modifications for transparency. At the end of the study, the dataset was archived securely for future use. This systematic approach ensures data accuracy, confidentiality, and accessibility throughout the research process.

Data analysis

Data were evaluated using the statistical program Statistical Product and Service Solutions (SPSS, version 23.0; IBM SPSS Statistics for Windows, Armonk, NY). Descriptive statistics (number, percentage, mean ± sd, median (min-max) were used. Student's T-test was used for univariate continuous data, and a chi-square test was used for categorical data. Logistic regression analysis was used in multivariate analysis. For the analysis, a type 1 error value of p<0.05 was considered significant.

## Results

As shown in Table 1, the average age of the mothers who participated in our study was 29.5 years, the ages vary with a standard deviation of 19.2 months. The proportion of mothers aged 29 years and over was 52.7%. The average age of the children is 29.4 months, the ages vary with a standard deviation of 5.3 months. Additionally, 50.8% of the children were 30 months of age or younger. Of the mothers, 96.9% were married, 3.1% were divorced or single; the mean age at first marriage was 22.5± 3.4 years, and 58.9% were married between the ages of 19-24 years. The mean age of the fathers of the children included in the survey was 32.6 ± 5.7 years (the minimum age was 21 years and the maximum age was 53 years for fathers). Whereas 4.7% of the mothers were illiterate, 37.6% had primary or secondary school education and 57.8% had high school or higher education. In contrast, 9% of the fathers were illiterate, 22.5% had primary or secondary school education, and 75.6% had high school or higher education. The rate of mothers who were employed in an income-generating job was 29.5%, and the rate of those who were not employed was 70.5%.

**Table 1:** Sociodemographic Characteristics SD = Standard Deviation

Variable																																																															Number of people							%				Mean	SD	Median	Range
FHC																																																																													
Yunusemre 12th family doctor																																																															130							50.4							
Sehzadeler 4th family doctor																																																															128							49.6							
Mother’s age (years)																																																																										29.5	5.4		
29 and under																																																															136							52.7							
30 and above																																																															122							47.3							
Child’s age (months)																																																																										29.4	19.2		
Under 30 months																																																															131							50.8							
30 months and above																																																															127							49.2							
Marital status																																																																													
Married																																																															250							96.9							
Single/divorced																																																															8							3.1							
Age at first marriage (years)																																																																										22.5	3.4		
18 years and under																																																															27							10.5							
19-24																																																															152							58.9							
25 years and older																																																															79							30.6							
Level of education																																																																													
Illiterate																																																															12							4.7							
Primary School - Secondary School																																																															97							37.6							
High school and above																																																															149							57.8							
Spouse’s level of education																																																																													
Illiterate																																																															5							1.9							
Primary School - Secondary School																																																															58							22.5							
High school and above																																																															195							75.6							
Employment status																																																																													
Yes																																																															76							29.5							
No																																																															182							70.5							
Social class																																																																													
Lower social class																																																															199							77.1							
Upper social class																																																															59							22.9							
Family type																																																																													
Nuclear family																																																															242							93.8							
Extended family (more than 2 generations live together)																																																															10							3.9							
Broken family																																																															6							2.3							
Perceived income																																																																													
Income less than expenditure																																																															97							37.6							
Income equal to expenditure																																																															123							47.7							
Income more than expenditure																																																															38							14.7							
Health insurance																																																																													
National social security / private insurance																																																															225							87.2							
On government benefits / none																																																															33							12.8							
Migration status																																																																													
Migrant																																																															90							34.9							
Non-migrant																																																															168							65.1							
If migrant, years since migration																																																																												6	1-35
Less than 10 years ago																																																															61							67.8							
More than 10 years ago																																																															29							32.2							
If migrant, region migrated from																																																																													
Western regions (Aegean, Marmara, Mediterranean, Black Sea)																																																															28							31.1							
Central Anatolia																																																															6							6.7							
Eastern regions (Eastern Anatolia, Southeastern Anatolia, Syria)																																																															56							62.2							

Of the families participating in our study, 77.1% were in the lower social class group and 22.9% in the upper social class group. Further, 93.8% of the families are nuclear families, 3.9% are extended families, and 2.3% are fragmented families. When the participants were asked about their perceived income level, 37.7% stated that their income was less than their expenses, 47.7% stated that their income and expenses were equal, and 14.7% stated that their income was more than their expenses. In the context of insurance, 87.2% of the participants had SSI or private insurance, and 12.8% were on social security benefits or no social security. Additionally, 65.1% of the respondents had migrated from another region, 62.2% of the migrants came from Eastern regions (Eastern Anatolia, Southeastern Anatolia, or Syria), 31.1% from Western regions (Aegean, Marmara, Mediterranean or Black Sea), and 6.7% from Central Anatolia. Moreover, 67.8% of these migrations within 10 years and 32.2% more than 10 years ago (Table 1).

Table 2 shows that 22.5% of the mothers who participated in the study were obese, 40.7% were overweight, and 36.4% were normal weight according to their BMIs. Of the children participating in the study, 54.3% were boys and 45.7% were girls. In terms of WFA, 7.8% of the children were obese, 2.7% were underweight, and 89.5% were normal. In terms of HFA, 15.1% were stunted, 5.4% were tall, and 79.5% were normal. According to their WFH, 12% of the children were obese, 4.3% were wasted, and 67% were normal. Further, 6.6% of the children were born with less than normal weight, 9.3% were born with more than normal weight, and 84.1% were born with normal weight. When it came to the mode of delivery, 53.9% of the children were born by cesarean section, and 46.1% were born vaginally. The double burden of malnutrition, characterized by child malnutrition combined with an overweight or obese mother, was present in 15.9% of cases (Table 2).

**Table 2:** Child Growth-Development and Maternal Characteristics

Variable	N	%
Maternal Body Mass Index (BMI)		
Normal	94	36.4
Overweight	105	40.7
Obese	58	22.5
Sex of the child		
Male	140	54.3
Girl	118	45.7
Weight-for-Age (WFA)		
Underweight (under -2z)	7	2.7
Normal (-2z to 2z)	231	89.5
Overweight (over 2z)	20	7.8
Height-for-Age (HFA)		
Stunted (under -2z)	39	15.1
Normal (-2z to 2z)	205	79.5
Tall (over 2z)	14	5.4
Weight-for-height (WFH)		
Wasted (below -2z)	11	4.3
Normal (-2z to 2z)	201	67.0
Overweight (over 2z)	31	12.0
Underweight child and overweight or obese mother		
No double burden	241	93.4
Double burden	17	6.6
Stunted child and overweight or obese mother		
No double burden	225	87.2
Double burden	33	12.8
Child's birth weight		
Less than normal	17	6.6
Normal	217	84.1
More than normal	24	9.3
Mode of delivery		
Vaginal	119	46.1
Cesarean section	139	53.9
Double burden of malnutrition (child malnutrition + overweight/obese mother)		
Present	41	15.9
Absent	217	84.1

As shown in Table 3, 43.7% of the mothers who participated in our survey had one child, and 52.7% had two or more children. Of the children surveyed, 54.7% were first-born, and 45.3% were second-born or later. When the surveyed children were born, 1.2% of mothers were 18 years of age or younger, 88.4% of mothers were between 19 and 34 years of age, and 10.5% of mothers were 35 years of age or older. Moreover, 14.7% of the children were born as a result of unintended pregnancy. During pregnancy, 97.7% of mothers were followed up on by a healthcare professional, and 2.3% were not followed up on at all. Of the mothers who had follow-ups, 91.3% had four or more, while 8.7% had three or fewer. Only 11.2% of mothers smoked during pregnancy. Breastfed children made up 94.2% of those surveyed. Among breastfed children, 80.2% started breastfeeding within one hour, 16.9% between one hour and one day, and 2.9% after one day. Thirty percent of children received breast milk at 12 months and older, 46.1% between 6-11 months, and 23.9% at five months and younger. Additionally, 67.2% of children were exclusively breastfed for the first six months, 32.8% started supplementary foods in the first six months, 25.2% of the children started supplementary foods before four months, 13.6% between four and six months, 51.2% after six months, and 10.1% had not yet started because they were younger than six months at the time of the survey. Further, 94.6% of children were taking vitamin D supplements, and 86.4% of children were taking iron supplements prescribed by their family doctor. Among the children surveyed, only 7.8% had a chronic disease that was diagnosed by a medical professional. Regarding diarrhea episodes in the past year, 31.3% of children never experienced it, 32.1% had one episode, and 36.7% had two or more episodes, with an average of 1.3 episodes. The data on upper respiratory tract infections (URTIs) in the past year show that 19.3% of the children had no URTIs, 33.2% had one, and 47.5% had two or more (Table 3).

**Table 3:** Pregnancy and Child Care Characteristics SD = Standard deviation; N = Number of participants; URTI = Upper respiratory tract infection

Variable	N		%		Mean	SD
Number of children in the family						
Only child	122		47.3			
2 children and more	136		52.7			
Birth order of the child						
First	141		54.7			
Second or later	117		45.3			
Mother’s age at childbirth (years)					27.3	5.2
18 years and under	3		1.2			
19-34 years	228		88.4			
35 years and older	27		10.5			
Desired pregnancy						
Yes	220		85.3			
No	38		14.7			
Received prenatal care						
Yes	252		97.7			
No	6		2.3			
Iron supplementation						
Yes	223		86.4			
No	35		13.6			
Chronic disease						
Yes	20		7.8			
No	238		92.2			
Number of episodes of diarrhea the child has had in the last 1 year					1.3	
None	75		31.3			
1	77		32.1			
2 or more	88		36.7			
Number of URTIs the child had in the last 1 year					1.8	
None	47		19.3			
1	81		33.2			
2 or more	116		47.5			

Table 4 shows that price increases over the past year have significantly impacted access to staple foods for 87.6% of individuals. Additionally, 91.5% report that their purchasing power has decreased in the last five years. Accessing healthy food is a financial struggle for 56.6% of people sometimes, and 34.9% always. Processed foods are purchased sometimes by 65.1% of the respondents, with 20.5% buying them always and 14.3% never. When it comes to checking expiration dates, 48.4% always do so, 41.9% sometimes, and 9.7% never. The same percentages apply to reading ingredient lists on packaging. Regarding diet perception, 75.2% consider their diet healthy sometimes, 12.8% always, and 12.0% never. Physical activity levels show that 52.3% engage in it sometimes, 39.1% never, and 8.5% always. Compliance with the Mediterranean Diet Adherence Scale (MDAS) is categorized as non-compliant for 51.2% of individuals, acceptable for 31.4%, and a tight fit for 17.4% (Table 4).

**Table 4:** Healthy Eating Characteristics

Variable	N	%
Have price increases in the last year impacted access to staple food?		
Yes	226	87.6
No	32	12.4
How has your purchasing power changed in the last 5 years?		
Decreased	236	91.5
Unchanged	22	8.5
Is accessing healthy food a financial struggle?		
Never	22	8.5
Sometimes	146	56.6
Always	90	34.9
How often do you buy processed foods?		
Never	37	14.3
Sometimes	168	65.1
Always	53	20.5
Do you examine expiration dates?		
Never	25	9.7
Sometimes	108	41.9
Always	125	48.4
Do you read the ingredients list on packaging?		
Never	25	9.7
Sometimes	108	41.9
Always	125	48.4
Do you consider your diet healthy?		
Never	31	12.0
Sometimes	194	75.2
Always	33	12.8
Do you engage in physical activity?		
Never	101	39.1
Sometimes	135	52.3
Always	22	8.5
Mediterranean Diet Adherence Scale compliance		
Non-compliant (6 and below)	132	51.2
Acceptable compliance (7 and 8)	81	31.4
Strict compliance (9 and above)	45	17.4

Table 5 shows a significant association between the primary care provider where mothers are registered and their weight status (P = 0.018). Among mothers registered at Sehzadeler No. 4, a semi-urban healthcare center, 70.3% are overweight or obese, compared to 56.2% at Yunusemre No. 12, an urban healthcare center. The mother’s education level shows a significant association with her weight status (P = 0.011). Among mothers with a high school education or above, 57.7% are overweight or obese. This prevalence increases to 68.0% among those with primary or secondary school education and jumps dramatically to 91.7% among illiterate mothers. Similarly, the spouse's education level is significantly associated with maternal weight (P = 0.016). Mothers with spouses who have a high school education or higher have a 59.0% prevalence of overweight or obesity, compared to 75.9% among those whose spouses have only primary or secondary education, and 80.0% among those with illiterate spouses. Health insurance type significantly affects maternal weight status (P = 0.002). Mothers with national health insurance or private insurance have a 59.6% prevalence of overweight or obesity, while this prevalence rises to 87.9% among those on social security benefits or without any health assurance. The consumption of packaged and processed foods is significantly linked to maternal overweight and obesity (P = 0.038). Among mothers who never consume these foods, 60.6% are overweight or obese, while 78.4% of those who sometimes or always consume these foods fall into this category.

Perception of healthy eating: Mothers’ perception of their diet also shows a significant association with their weight (P = 0.032). Among those who never think they eat healthily, 60.8% are overweight or obese. This percentage rises to 80.6% among those who believe they sometimes or always eat healthily. Physical activity levels are significantly associated with maternal weight (P = 0.015). In terms of physical activity, 42.7% of individuals never engage in it, compared to 27.7% who do so sometimes or anytime. Adherence to the Mediterranean diet is strongly associated with maternal weight status (P < 0.00). Mothers who are not compliant with the diet (scoring 6 or below) have a 72.0% prevalence of overweight or obesity. Among those with acceptable adherence (scoring 7-8), 63.0% are overweight or obese. Interestingly, strict adherence to the diet (scoring 9 and above) is associated with a significantly lower prevalence of overweight or obesity at 37.8%. The perceived income level of the household significantly impacts maternal weight status (P = 0.007). Mothers whose income is less than their expenses have a 71.1% prevalence of overweight or obesity, compared to 63.4% among those whose income equals their expenses. Interestingly, only 42.1% of mothers whose income exceeds their expenses are overweight or obese, indicating that financial stability may be linked to better weight management. The presence of chronic disease in mothers is significantly associated with higher rates of obesity (P = 0.029). Among those with chronic diseases, 78.9% are overweight or obese, compared to 60.5% of mothers without chronic diseases. Smoking during pregnancy is another factor significantly associated with maternal overweight and obesity (P = 0.014). Among mothers who smoked during pregnancy, 82.8% are overweight or obese, compared to 60.7% among non-smokers. Economic difficulties in accessing healthy food are strongly associated with maternal overweight and obesity (P = 0.001). Among mothers who always face economic difficulties, 76.7% are overweight or obese. This prevalence decreases to 56.8% among those who sometimes face difficulties and further to 50.0% among those who never have difficulties accessing healthy food (Table 5).

**Table 5:** Variables Associated With Maternal Overweight and Obesity P < 0.05 was considered significant; x^2 ^= chi-squared test values against the null hypothesis H_0_

Variable	Weight of the mother							P	X^2^
	Low Weight-Normal			Overweight-Obesity					
	N		%	N			%		
Which primary care provider they’re registered at								0.018	38.492
Sehzadeler No. 4 (semi-urban)	38	29.7			90	70.3			
Yunusemre No. 12 (urban)	57	43.8			73	56.2			
Education level of the mother									
High School and Above	63	42.7			86	57.7		0.011	39.455
Primary School Secondary School	31	32.0			66	68.0			
Illiterate	1	8.3			11	91.7			
Education level of the spouse									
High School and Above	80	41.0			115	59.0		0.016	38.835
Primary School Secondary School	14	24.1			44	75.9			
Illiterate	1	20.0			4	80.0			
Health Insurance									
National / private	91	40.4			134	59.6		0.002	41.250
On social security benefits / none	4	12.1			29	87.9			
Packaged and processed food consumption									
Never	87	39.4			134	60.6		0.038	37.691
Sometimes / always	8	21.6			29	78.4			
Do you think you eat healthy								0.032	37.900
Never	89	39.2			138	60.8			
Sometimes / always	6	19.4			25	80.6			
Physical Activity								0.015	38.712
Never	67	42.7			90	57.3			
Sometimes / always	28	27.7			73	72.3			
Mediterranean Diet Adherence Scale								˂0.00	45.637
Non-compliant (6 and Below)	37	28.0			95	72.0			
Acceptable compliance (7-8 Points)	30	37.0			51	63.0			
Strict compliance (9 and Above)	28	62.2			17	37.8			
Getting Spouse's Permission to Go Out								0.014	38.787
Yes	21	25.9			60	74.1			
No	74	41.8			103	58.2			
Economic Difficulty in Accessing Healthy Food								0.001	42.053
Always	21	23.3			69	76.7			
Sometimes	63	43.2			83	56.8			
Never	11	50.0			11	50.0			

Table 6 examines the relationship between the frequency of diarrhea and the occurrence of wasting (low WFH) in children. Wasting is indicated by a WFH z-score of -2 or below, while children who are normal or overweight have a z-score of 2 or above. Among children who did not experience diarrhea, only 1.3% are wasted, while a significant 98.7% are normal or overweight. This suggests that the absence of diarrhea is strongly associated with a lower prevalence of wasting. For children who had diarrhea once, 9.1% are wasted, while 90.9% are normal or overweight. The higher percentage of wasted children compared to those with no diarrhea indicates a possible link between even a single episode of diarrhea and an increased risk of wasting. Among children who experienced two or more episodes of diarrhea, 2.3% are wasted, and 97.7% are normal or overweight. Notably, despite the increased frequency of diarrhea, the percentage of wasted children is lower than those with just one episode, but still higher than those with no diarrhea. The P-value of 0.031 indicates that there is a statistically significant association between the frequency of diarrhea and the likelihood of a child being wasted (Table 6).

**Table 6:** Conditions Associated With Having a Wasted Child (WFH) P < 0.05 was considered significant.

Variable	Weight-For-Height(WFH)				P
	Wasted (-2z and below)		Normal/overweight (2z and above)		
	N	%	N	%	
Diarrhea					0.031
No diarrhea	1	1.3	74	98.7	
1 episode of diarrhea	7	9.1	70	90.9	
2 or more episodes of diarrhea	2	2.3	86	97.7	

Table 7 highlights several conditions associated with low birth weight (LBW) in children, considering both underweight and normal/fat categories. Younger children, under 30 months, show a higher percentage of being underweight (3.8%) compared to those 30 months and older (1.6%), with the majority in both groups falling into the normal/fat category. Income levels also influence weight, with a slightly higher percentage of underweight children in households where income is less than expenditure (3.1%), compared to those with income equal to (2.4%) or greater than expenditure (2.6%), although the majority remain in the normal/fat category across all income levels. Iron supplementation significantly impacts weight outcomes, with only 2.2% of children taking supplements being underweight, compared to 5.7% of those who do not. Family size also shows a correlation, as children in single-child families are less likely to be underweight (2.1%) compared to those in larger families with two or more children (3.4%) (Table 7).

**Table 7:** Conditions Associated With Low Birth Weight (LBW) of the Child P < 0.05 was considered significant.

Variable	Weight by Age				P
	Underweight (-2z and under)		Normal and Fat		
	N	%	N	%	
Child Age					0.032
30 months and above	2	1.6	125	98.4	
Under 30 months	5	3.8	126	96.2	
Perceived Income					0.046
Income less than expenditure	3	3.1	94	96.9	
Income equal to expenditure	3	2.4	120	97.6	
Income more than expenditure	1	2.6	37	97.4	
Taking Iron Supplements					0.002
Yes	5	2.2	218	97.7	
No	2	5.7	33	94.3	
Child's Birth Order					
First child	3	2.1	138	97.8	0.046
Second child or later	4	3.4	113	96.6	

Table 8 shows that, among households with fathers having a high school education or higher, 11.3% had a double burden, and 88.7% did not experience it. Among those with primary or secondary school education, 29.3% experienced a double burden, while 70.7% did not. For illiterate fathers, 40.0% had a double burden, whereas 60.0% did not experience it. In upper social class households, 5.1% had a double burden, and 94.6% did not experience it. Among lower social class households, 19.1% had a double burden, while 80.9% did not experience it. Households with mothers who had private or SSI health insurance showed a double burden in 13.3% of cases, while 86.7% did not. Conversely, in households where mothers had no health insurance or were on social security benefits, 33.3% had a double burden, while 66.7% did not experience it. Households migrating from Central Anatolia showed a double burden in 33.3% of cases, while 66.7% did not experience it. Among those from the Eastern-Southeastern-Syria regions, 25.0% had a double burden, and 75.0% did not experience it. In households migrating from the Aegean-Marmara-Mediterranean-Marmara-Black Sea regions, 3.6% had a double burden, while 96.4% did not experience it. Households, where the surveyed child's birth order was two or above, experienced a double burden in 21.4% of cases, while 78.6% did not experience it. Meanwhile, in households where the child's birth order was one, 11.3% had a double burden, while 88.7% did not experience it. Among mothers who reported sometimes/always consuming packaged and processed foods, 27.0% had a double burden, and 73.0% did not experience it. For mothers who selected "never" in response to the same question, 14% experienced a double burden, while 86% did not experience it. Households affected by price increases in the last year saw a double burden in 17.7% of cases, while 82.3% did not experience it. In households not affected by price increases, 3.1% had a double burden, while 96.9% did not experience it. Among surveyed children, 13.2% born from intended pregnancies had a double burden, while 86.8% did not experience it. In contrast, 31.6% of children born from unwanted pregnancies had a double burden, while 68.4% did not experience it. The data indicates that price increases in the last year have significantly impacted the ability to purchase staple foods for 82.3% of individuals, compared to 17.7% who were not affected (Table 8).

**Table 8:** Double Burden of Malnutrition and Its Associated Factors P < 0.05 was considered significant; x^2^=chi squared test values against the null hypothesis H_0_

Variable	No Double Burden		Double Burden		P	X^2^
							Number (N)	Percentage (%)	Number (N)	Percentage (%)
	Education level of the spouse						0.000	184.595
High school education and above	173	88.7	22	11.3				
Primary-secondary school	41	70.7	17	29.3				
Illiterate	3	60.0	2	40.0				
Social Class					0.10	183.578
Upper Social class	56	94.9	3	5.1		
Lower social class	161	80.9	38	19.1		
Health insurance					0.002	183.884
National health insurance /private insurance	195	86.7	30	13.3		
Income-based social health benefits or none	22	66.7	11	33.3		
Migration region					0.021	60.433
Western regions (Aegean, Marmara, Mediterranean, Black Sea)	27	96.4	1	3.6		
Central Anatolia	4	66.7	2	33.3		
Eastern regions (Eastern Anatolia, Southeastern Anatolia, Syria)	42	75.0	14	25.0		
Birth order of the child					0.028	183.279
First	125	88.7	16	11.3		
Second or other	92	78.6	25	21.4		
Packaged and processed food consumption					0.045	183.152
Never	190	86.0	31	14.0		
Sometimes/always	27	73.0	10	27.0		
Mediterranean diet compliance					0.012	183.761
Non-compliant (6 points and below)	106	80.3	26	19.7		
Acceptable compliance (7-8)	67	82.7	14	17.3		
Strict compliance (9 and above)	44	97.8	1	2.2		
Family type					0.008	183.641
Nuclear family	207	85.5	35	14.5		
Extended Family	7	70.0	3	30.0		
Broken family	3	50.0	3	50.0		
Perceived income					0.000	184.814
Income less than expenditure	71	73.2	26	26.8		
Income equal to expenditure	110	89.4	13	10.6		
Income more than expenditure	36	94.7	2	5.3		
Child birth weight					0.001	184.618
Less than normal	10	58.8	7	41.2		
Normal	190	87.6	27	12.4		
More than usual	17	70.8	7	29.2		
Desired pregnancy					0.004	183.819
Yes	191	86.8	29	13.2		
No	26	68.4	12	31.6		
Price increase in the last 1 year affecting the purchase of staple foods					0.035	183.223
Yes	186	82.3	40	17.7		
No	31	96.9	1	3.1		

Table 9 shows the multivariate regression analysis and identifies significant factors associated with the double burden of malnutrition at the household level. The birth order of the child plays a crucial role; families with a second or subsequent child are 2.25 times more likely to experience this burden compared to those with a first child (CI: 1.07-4.72). Additionally, the type of health insurance coverage impacts the likelihood of facing a double burden. Households with no health insurance or relying on social security benefits are 2.59 times more likely to experience a double burden of malnutrition compared to those with national or private health insurance (CI: 1.06-6.28). These findings highlight the importance of both birth order and health insurance coverage in understanding the factors contributing to the double burden of malnutrition in households (Table 9).

**Table 9:** Assessment of the Double Burden of Malnutrition at the Household Level and Associated Factors via the Logistic Regression-Reduced Final Model OR = Odds ratio; CI = Confidence Interval *Variables included in the model: Spouse's education, Social class, Health insurance, Migration region, Birth order, Packaged and processed food consumption, Mediterranean type diet, Family type, Perceived income, Child's birth weight, Desired pregnancy, Effect of price increase in basic food items on purchasing power in the last year **Backward Wald

	The double burden of malnutrition*	
Variable	P**	OR (95% CI)
Birth order		
First child	0.031	1 (Ref)
2nd and other child		2.25 (1.07-4.72)
Health insurance		
National health insurance- private	0.035	1 (Ref.)
On social security benefits - none		2.59 (1.06-6.28)

The logistic regression analysis in Table 10 highlights significant factors associated with stunting (HFA) in children. Age is a critical factor, with children over 30 months being 5.57 times more likely to experience stunting compared to those 30 months and below (CI: 2.22-13.94). Birth weight also plays a role; children born with less than normal weight are 9.32 times more likely to be stunted compared to those born with more than usual weight (CI: 1.24-70.07). Additionally, breastfeeding has a protective effect; children who were not breastfed are 6.80 times more likely to be stunted compared to those who were breastfed (CI: 1.93-23.89). These findings underscore the importance of child age, birth weight, and breastfeeding in influencing the likelihood of stunting (Table 10).

**Table 10:** Evaluation of Stunting (HFA) and Associated Factors According to the Logistic Regression-Reduced Final Model OR = Odds ratio; CI = Confidence interval *Variables included in the model: Age of the child, Perception of healthy nutrition, Birth weight of the child, Desired pregnancy, Breastfeeding status, Mother's ability to make self-determination **Backward Wald

Stunting*		
Variable	P**		OR (95% CI)
Child age			
30 months and below	0.000	1 (Ref)	
Over 30 months		5.57 (2.22-13.94)	
Birth weight			
More than usual	0.030	1 (Ref)	
Normal		2.62 (0.50-13.77)	
Less than normal		9.32 (1.24-70.07)	
Breastfeeding			
Yes	0.003	1 (Ref)	
No.		6.80 (1.93-23.89)	

The logistic regression analysis in Table 11 identifies a significant association between birth order and the likelihood of a child being underweight. Specifically, children who are second-born or later are 3.17 times more likely to be underweight compared to first-born children (95% CI: 1.29-7.82) (Table 11).

**Table 11:** Evaluation of Underweight (WFA) and Associated Factors According to the Logistic Regression-Reduced Final Model OR = Odds ratio; CI = Confidence interval *Variables included in the model: Child's age, Birth order, Perceived income, Iron used as a supplement **Backward Wald

	Underweight*		
Variable	P**		OR (95% CI)
Birth order			
First child		0.012	1 (Ref)
2 and above			3.17 (1.29-7.82)

Table 12 highlights the factors associated with maternal obesity or overweight by logistic regression analysis. The presence of health insurance plays a significant role, with mothers without health insurance or relying on social security benefits being 4.70 times more likely to be obese or overweight compared to those with national health insurance (CI: 1.53-14.40). Consumption of packaged and processed foods also affects obesity risk; mothers who consume these foods sometimes or always are 4.21 times more likely to be obese or overweight compared to those who never consume them (CI: 1.63-10.85). Physical activity levels are linked to obesity risk as well, with mothers who never engage in physical activity being 2.17 times more likely to be obese or overweight compared to those who are always active (CI: 1.19-3.94). Mediterranean diet compatibility shows that mothers with acceptable diet compliance are 3.68 times more likely to be obese or overweight than those fully compliant with the diet (CI: 1.63-8.33), and non-compliance is associated with a 2.73 times higher risk (CI:1.19-6.30). Additionally, smoking during pregnancy increases the likelihood of obesity or overweight in mothers by 3.24 times compared to non-smokers (CI: 1.12-9.35) (Table 12).

**Table 12:** Evaluation of Factors Associated With Maternal Obesity or Overweight According to the Logistic Regression-Reduced Final Model OR = Odds ratio; CI = Confidence interval *Variables included in the model: FHC number, Educational status, Spouse's educational status, Health insurance, Packaged and processed food use, Thinking that they are eating healthy, Physical activity, FAS, Perceived income, Presence of chronic disease, Smoking status during pregnancy, Getting permission from spouse when going out, Having economic difficulties in accessing healthy foods **Backward Wald

Obese or Overweight Mother*		
Variable	P**		OR (95% CI)
Health insurance			
National health insurance		0.006	1 (Ref)
None or on social security benefits			4.70 GA (1.53-14.40)
Packaged and processed Food Consumption			
Never		0.002	1 (Ref)
Sometimes-always			4.21 GA (1.63-10.85)
Physical Activity			
Always		0.006	1 (Ref)
Sometimes			2.52 (0.86-7.35)
Never			2.17 GA (1.19-3.94)
Mediterranean diet adherence score			
Full compliance with the diet		0.002	1 (Ref)
Acceptable fit			3.68 (1.63-8.33)
Non-compliant with diet			2.73 GA (1.19-6.30)
Smoking during pregnancy			
No		0.03	1 (Ref)
Yes			3.24 GA (1.12-9.35)

## Discussion

Over the past 20 years, significant progress has been made in enhancing maternal and child nutrition, with a notable reduction of one-third in the prevalence of stunted growth among children. However, the triple burden of malnutrition - stunting, wasting, and overweight - continues to threaten children's overall well-being. Stunting describes a condition where a child is shorter than expected for their age. It can lead to serious and irreversible physical and cognitive impairments. Child wasting is a dangerous condition caused by inadequate nutrient intake or frequent illnesses. Children affected by wasting have weakened immune systems, are prone to long-term developmental delays, and are at a higher risk of death, especially in severe cases. Childhood overweight occurs when a child's calorie intake surpasses their energy needs. This type of malnutrition is fueled by the limited access to affordable, nutritious foods, combined with the widespread marketing of nutrient-poor, ultra-processed products.

According to the 2017 Turkey Nutrition and Health Survey, 27.6% of the female population in Turkey is overweight, and 39.1% is classified as obese; for children aged 15-18, 4.6% were stunted, with boys at 5.1% and girls at 4.2% [5]. The prevalence of severely underweight children, based on BMI, was 2.6% (1.5% for boys and 3.8% for girls), while 15.6% were underweight (16.6% for boys and 12.0% for girls) [5]. Additionally, obesity prevalence was 8.3% (8.3% for boys, 8.2% for girls), and overweight prevalence was 13.3% (13.5% for boys, 13.0% for girls) [5].

This study was conducted among women with children aged 0-5 years who attended the semi-urban Sehzadeler FHC No. 4 and urban Yunusemre FHC No. 12 in the province of Manisa. Specifically, 15.1% of the children were found to be stunted (HFA), 2.7% underweight (WFA), 7.8% overweight, and 12.0% obese. Among the mothers who participated in our study, 63.2% were found to be overweight and obese. The frequency of double burden of malnutrition was found to be 15.9% at the household level. In the univariate analyses, it was determined that, being in a lower social class, having social security health benefits or not, having a broken family structure, having a smaller birth weight than normal, having a low perceived income, the father being uneducated, and undesired pregnancy corresponded with increased rates of DBM. It was also found that households frequently consuming packaged foods and experiencing economic challenges in accessing nutritious food in recent years significantly contributed to the risk of the double burden of malnutrition. Multivariate analysis revealed that additional factors, such as being the second-born child or later and the family lacking social security enrollment, were associated with an increased risk of the double burden of malnutrition.

Our univariate analyses revealed that low spousal education, being in a lower social class, lack of health insurance, migrating from Central Anatolia, being second or later in the birth order, consuming packaged food, non-compliance with the Mediterranean diet scale, having a broken family, having low perceived income, having a low birth weight, having an unintended pregnancy, and having reduced access to basic nutrients in the last one year were found to be significantly associated with the double burden of malnutrition.

Multivariate analysis disclosed birth order of two or more increases the likelihood of DBM 2.2-fold (95% CI: 1.07-4.7), and lack of health insurance increases the probability of DBM 2.5-fold (95% CI: 1.06-6.28). The prevalence of stunting was found to be 15.1%. Our multivariate analysis uncovered that children over 30 months have an increased prevalence of stunting by 5.5 times (95% CI: 2.2-13.9), birth weight less than normal increased its prevalency 9.3 times (95% CI: 1.2-70.07), not receiving breast milk increased its prevalence 6.8 times (95% CI: 1.9-23.8), and mother's lack of participation in household decisions increased its prevalence 2.3 times (95% CI: 1.06-5.1). In our study, the prevalence of underweight subjects was found to be 10.5%. Multivariate analysis revealed the risk of underweight increases 3.1 times (95% CI: 1.29-7.82) if the birth order is second or later. The prevalence of the mother being obese or overweight is 63.2%. Multivariate analysis indicated that the lack of health insurance of the mother increases the risk of DBM 4.7 times (95% CI: 1.53-14.40), consumption of packaged food 4.2 times (95% CI: 1.63-10.85), lack of physical activity of the mother 2.1 times (95% CI: 1.19-3.94), non-compliance with the Mediterranean diet 2.7 times (95% CI: 1.19-6.30), and smoking during pregnancy 3.2 times (95% CI: 1.12-6.35).

A significant link was found between the educational level of the partners of the women participating in our study and the double burden of malnutrition. The double burden of malnutrition was found significantly more often in the households of women with illiterate spouses. A significant correlation between low education levels in the spouse and the double burden of malnutrition was also shown in the 2006 Brazilian demographic and health survey [27]. Per these results, it can be stated that as the father's education level increases, malnutrition in his children decreases and thus the double burden in the household will decrease.

We found a positive correlation between household income level and the double burden of malnutrition. The DBM had a higher incidence rate in lower social class households which decreased in households with higher income levels. In a 2007 study conducted in Mexico, similar trends were also reported [28]. The absence of health insurance, another income-level indicator, was significantly associated with the double burden of malnutrition during our analysis. On the other hand, a study conducted in India in 2016 observed that the prevalence of DBM was higher in families in the upper social class [29].

One of the most prominent risk factors for malnutrition is the household income level [30]. The level of food consumption is closely related to income level. Studies have shown that families with low-income levels have a more limited supply of basic nutrients [31], and it has been found that the incidence of malnutrition is higher in children of families with low-income levels [32].

In our survey, 87.6% of the women surveyed said that price increases in the last year had affected their intake of basic nutrients. That said, malnutrition rates are not very high. The fact that malnutrition is a chronic condition that takes time to appear and that the children participating in the survey were between the ages of zero and five years could explain its low incidence. Despite this, a significant correlation was found between basic food purchasing power and the double burden of malnutrition. This result was also found to be significant in similar studies [33].

We found a positive correlation between family structure and DBM. The double burden was observed more frequently in broken families. In a 2012 study conducted in Indonesia and Bangladesh, it can be seen that extended families increased the double burden [34]. In this study conducted in Manisa, women belonging to broken families were more likely to report low perceived income levels.

Therefore, we postulate that family structure may have affected DBM rates indirectly through perceived income level. In the multivariate analysis, family type and income level did not maintain their statistical significance. When we interpreted the results of our study together with the literature, we saw that the causality of DBM was multifactorial.

The region the families who participated in our study migrated from significantly influenced the presence of the double burden of malnutrition at the household level. The rate of DBM was higher in those who migrated from Central Anatolia compared to those who migrated from eastern and western regions. The 2010 and 2019 data of the TBSA manifested that Central Anatolia was found to have the highest prevalence of both overweight and obesity in women and of underweight, overweight, and obesity in children aged 0-5 years, while northeast Anatolia had the highest prevalence of stunting [35,36]. Our study’s results were consistent with other literature in this area.

LBW remains a major public health problem in many developing countries, and malnutrition both before and during pregnancy is recognized as a major cause [37]. A randomized controlled trial conducted in Gambia in 1997 with 379 pregnant women showed that prenatal high-energy dietary supplementation significantly reduced intrauterine growth retardation and perinatal mortality [38]. In our study, 41.2% of the children of overweight mothers were born with low birth weight, which may be explained by maternal consumption of foods high in calories but low in nutrients.

Babies born with a low birth weight are at risk of developing type 2 diabetes and cardiovascular disease in adulthood [39]. Many studies have found that these diseases are a direct contributor to adult obesity [40]. A 1998 study conducted in the Cape Peninsula showed that a large proportion of malnourished children went on to become overweight [13]. In a study conducted in Guatemala, significant differences were found in the birth weight of the next generation depending on the type of supplementation that mothers were exposed to in their first year of life; mothers themselves born with low birth weight were more likely to have children born with low birth weight [41]. This suggests that intergenerational cultural transmission may play a key role in malnutrition.

In this vein, a study published in the American Journal of Clinical Nutrition in 2004 concluded that women's nutrition and health should be continuously invested in throughout their lives, especially to prevent low birth weight and related chronic diseases in the future and to prevent intergenerational malnutrition [37]. In our study, 35.3% of stunted children had a low birth weight. In a study from Indonesia, the main determinant of stunted children was low birth weight. The same study found that the probability that these babies would be stunted was 1.74 times higher than babies born at normal weight [42].

Another variable associated with the double burden of malnutrition was whether the pregnancy was desired. DBM was significantly higher in unintended pregnancies, but this variable was not significant in logistic regression analysis. In developing countries, it is estimated that approximately 40% of pregnancies are unwanted or mistimed, and half of these are terminated, with only 38% reaching term [43]. Some studies have found that overweight and obese women are more likely to have an unintended pregnancy than women with normal BMI [44,45]. A sexual behavior survey conducted in France in 2016 revealed that obese women under 30 years of age were less likely to seek health services for contraception or use oral contraceptives, but more likely to report an unintended pregnancy [46].

In a review of data from the 2011 Bangladesh demographic and health survey, 39% of wanted children and 46% of unwanted children were stunted [47]. A study conducted in India in 2012 found that children from unwanted pregnancies were more likely to be unvaccinated and stunted than wanted children [48]. The mother's feelings towards the unwanted child may lead to conscious or unconscious neglect of the child and subsequent malnutrition due to the weak mother-infant bond. Easy access to family planning methods, especially for mothers with low socioeconomic status, could contribute to a decrease in the population of stunted children.

The nutritional profile of the family’s diet was also surveyed in this study. Nutrition-related variables associated with the double burden of malnutrition were packaged food consumption and access to basic nutrients in the last one and five years, respectively. Frequent use of packaged foods in the family and difficulty in accessing staple foods in the last year were found to be significantly positively correlated with the incidence of DBM in the household. There was no significant correlation between purchasing power in the last five years and household DBM.

Data from Euromonitor International show increasing trends in sales of non-nutritive junk food and sugar-sweetened beverages in Chile, South Africa, the Philippines, and Malaysia [49]. In developing countries, where the double burden of malnutrition is a particularly pronounced problem, urbanization, migration to cities, income growth, infrastructure improvements and liberalization of global trade policy have encouraged private investment in packaged food production [50]. At the same time, the increase in the number of women working outside the home has increased the demand for packaged ready-to-eat food [51]. Monteiro et al. called this increasing shift in food preparation and consumption the ultra-processed food revolution [52]. In a study conducted by Popkin et al. in low- and middle-income countries, it was concluded that the consumption of packaged foods increased with this changing trend in nutrition and that this situation directly affected the double burden of malnutrition [8]. In our study, conducted in Manisa, the double burden of malnutrition was significantly higher in households where the packaged food consumption question was answered as sometimes/always.

Another variable questioned in the study related to nutrition was the MDAS. According to the MDAS, 19.7% of households with poor dietary adherence had a double burden, while 17.3% of households with acceptable dietary adherence had a double burden. The rate of double burden in households with strict dietary adherence was 2.2%.

In a study in which diet quality was compared and associated with the double burden of malnutrition, adherence to the Mediterranean diet, BMI, waist circumference, and weight loss were found to be insignificant for adults; Mediterranean Diet Quality Index (KIDMED - adherence to the Mediterranean diet in children) and BMI were found to be insignificant to waist circumference and overweight or obesity in children [53]. The reason for the divergence of these studies could be attributed to the differences in sociocultural and physical activities of the regions.

Another factor affecting DBM in the household is the birth order of the child. Children second and later in the birth order have 2.2 (1.07-4.7) times higher risk of DBM in the household. Comparable results from Kayseri, Turkey, determined that second- and third-born children were more likely to be underweight than first-born children [54]. In 2012 studies conducted in Bangladesh and Indonesia, a relationship was found between birth order and the double burden of malnutrition [34]. It is considered that birth order in children indirectly depends on the maternal education and income status of the family. In similar studies, it has been shown that the number of children is higher in families with low levels of maternal education and income [55].

In this study, the prevalence of underweight (BGA) was found to be 4.3%, with a significant correlation to a high prevalence of diarrhea discovered in univariate analysis. The fact that the study was conducted during the fall may have affected the rate of malnutrition due to the increase in diarrhea and respiratory tract infections around this time of year.

Another issue that should be emphasized in this study is the rate of overweight and obese mothers, which sits at 63.2%. Our multivariate analysis showed that high consumption of packaged foods, physical inactivity, lack of health insurance, smoking during pregnancy, and non-compliance with the Mediterranean diet were variables associated with mothers being overweight and obese.

Limitations

The reality that the study was conducted on only those individuals who attended the family health clinics and agreed to participate prevents the results from being more broadly generalizable. Determining the socioeconomic status of individuals, maternal self-determination, migration status, birth weight of children, family structure, pregnancy, and breastfeeding is also based on self-reported data. This can itself be biased. Despite these limitations, there are ample strengths of this study. The core value of our study is that it offers a new perspective in terms of assessing malnutrition at the household level. We could not find any other study in our literature search on the double burden of malnutrition in Turkey, so our research is novel. In this respect, our study contributes a new source to this area of literature. The fact that body weight and height measurements of children and mothers were made by the researchers themselves, and not self-reported or taken by others, is an advantage for data reliability and quality purposes.

## Conclusions

In our study conducted among mothers at Manisa Şehzadeler FHC No. 4 and Yunusemre FHC No. 12, the double burden of malnutrition was identified in 15.9% of households. Multivariate analyses revealed that having two or more children increases the burden 2.2-fold (95% CI: 1.07-4.7), and lacking health insurance increases it 2.5-fold (95% CI: 1.06-6.2). The prevalence of overweight/obesity among mothers, at 63%, exacerbates the issue, with consumption of packaged foods elevating maternal obesity risk by 4.2 times (95% CI: 1.6-10.8). Our recommendations include educating mothers on the risks of packaged foods, advocating for legislative measures to improve food content, enhancing family planning services, and addressing difficulties in accessing staple foods. Additionally, with stunting rates at 15.1%, exceeding the national average, local authorities should provide targeted food aid to those most in need. Expanding health coverage to all citizens could alleviate the double burden of malnutrition, particularly for those without insurance.

## References

[1] Önal S, Özdemir A, Meşe C, Koca Özer B (2016). [Evaluation of the prevalence of obesity and malnutrition in preschool children: the case of Ankara]. DTCF Dergisi.

[2] Ersoy B, Günay T, Günes HS (2007). [Stunting in primary school children and its association with obesity]. Turkiye Klinikleri J Pediatr.

[3] (2024). UNICEF-WHO-the World Bank: joint child malnutrition estimates (JME) — levels and trends - 2023 edition. https://data.unicef.org/resources/jme-report-2023/.

[4] (2024). The state of the world’s children 2009-2013. https://www.unicef.org/reports/state-of-worlds-children.

[5] Republic of Turkey Ministry of Health (2019). Turkey Nutrition and Health Survey 2017 (TNHS).

[6] (2024). Obesity and overweight. https://www.who.int/en/news-room/fact-sheets/detail/obesity-and-overweight.

[7] Turkish Statistical Institute (2024). Türkiye health survey, 2019. Turkey Health Survey.

[8] Popkin BM, Corvalan C, Grummer-Strawn LM (2020). Dynamics of the double burden of malnutrition and the changing nutrition reality. Lancet.

[9] Wells JC, Sawaya AL, Wibaek R, Mwangome M, Poullas MS, Yajnik CS, Demaio A (2020). The double burden of malnutrition: aetiological pathways and consequences for health. Lancet.

[10] Doak CM, Adair LS, Bentley M, Monteiro C, Popkin BM (2005). The dual burden household and the nutrition transition paradox. Int J Obes (Lond).

[11] Barquera S, Peterson KE, Must A (2007). Coexistence of maternal central adiposity and child stunting in Mexico. Int J Obes (Lond).

[12] Kimani-Murage EW, Muthuri SK, Oti SO, Mutua MK, van de Vijver S, Kyobutungi C (2015). Evidence of a double burden of malnutrition in urban poor settings in Nairobi, Kenya. PLoS One.

[13] Steyn K, Bourne L, Jooste P, Fourie JM, Rossouw K, Lombard C (1998). Anthropometric profile of a black population of the Cape Peninsula in South Africa. East Afr Med J.

[14] Gillespie S, Haddad L (2001). Attacking the Double Burden of Malnutrition in Asia and the Pacific. Asian Development Bank.

[15] Haddad L, Cameron L, Barnett I (2015). The double burden of malnutrition in SE Asia and the Pacific: priorities, policies and politics. Health Policy Plan.

[16] Ramirez-Zea M, Kroker-Lobos MF, Close-Fernandez R, Kanter R (2014). The double burden of malnutrition in indigenous and nonindigenous Guatemalan populations. Am J Clin Nutr.

[17] (2024). Community-based management of severe acute malnutrition: a joint statement by the World Health Organization, the World Food Programme, the United Nations System Standing Committee on Nutrition, and the United Nations Children's Fund. World.

[18] Jamison DT (1986). Child malnutrition and school performance in China. J Dev Econ.

[19] Bayrı S, Egemen A (1984). [Malnutrition prevalence and affecting factors in rural areas]. Bes Diy Derg.

[20] Kar BR, Rao SL, Chandramouli BA (2008). Cognitive development in children with chronic protein energy malnutrition. Behav Brain Funct.

[21] Fischer L, Galler J (2015). Early childhood malnutrition increases metabolic syndrome in adulthood. FASEB J.

[22] Sutopa TS, Bari W (2022). How does mode of delivery associate with double burden of malnutrition among mother-child dyads?: a trend analysis using Bangladesh demographic health surveys. BMC Public Health.

[23] World Health Organization (2024). World Health Organization: A growth chart for international use in maternal and child health care: Guidelines for primary health care. A Growth Chart for International Use in Maternal and Child Health Care: Guidelines for Primary Health Care Personnel.

[24] (2024). A healthy lifestyle - WHO recommendations. https://www.who.int/europe/news-room/fact-sheets/item/a-healthy-lifestyle---who-recommendations.

[25] Pehlivanoğlu EF, Balcıoğlu H, Ünlüoğlu İ (2020). [Adaptation, validity and reliability of the Mediterranean diet adherence scale into Turkish]. OMJ.

[26] Boratav K (1993). Book review: state and class in Turkey. A study in capitalist development. Rev Radical Pol Econ.

[27] Castro IR, Anjos LA, Lacerda EM (2024). Brazil national population and health survey: children and women 2006-2007. Cad Saude Publica.

[28] Leroy JL, Habicht JP, González de Cossío T, Ruel MT (2014). Maternal education mitigates the negative effects of higher income on the double burden of child stunting and maternal overweight in rural Mexico. J Nutr.

[29] Patel R, Srivastava S, Kumar P, Chauhan S (2020). Factors associated with double burden of malnutrition among mother-child pairs in India: a study based on national family health survey 2015-16. Child Youth Serv Rev.

[30] Kikafunda JK, Walker AF, Collett D, Tumwine JK (1998). Risk factors for early childhood malnutrition in Uganda. Pediatrics.

[31] Başoğlu S, Besler T, Ciğerim N (1992). [Food expenditure shares of families depending on their socio-economic and income levels]. Bes Diy Derg.

[32] Narayan J, John D, Ramadas N (2019). Malnutrition in India: status and government initiatives. J Public Health Pol.

[33] Nugent R, Levin C, Hale J, Hutchinson B (2020). Economic effects of the double burden of malnutrition. Lancet.

[34] Oddo VM, Rah JH, Semba RD (2012). Predictors of maternal and child double burden of malnutrition in rural Indonesia and Bangladesh. Am J Clin Nutr.

[35] (2024). [Türkiye nutrition and health survey, 2010]. https://hsgm.saglik.gov.tr/depo/birimler/saglikli-beslenme-ve-hareketli-hayat-db/Dokumanlar/Kitaplar/TBSA_2017_Ozet_Bulgular.pdf.

[36] (2024). [Türkiye nutrition and health survey, 2019]. https://hsgm.saglik.gov.tr/depo/birimler/saglikli-beslenme-ve-hareketli-hayat-db/Dokumanlar/Kitaplar/TBSA_2017_Ozet_Bulgular.pdf.

[37] Ramakrishnan U (2004). Nutrition and low birth weight: from research to practice. Am J Clin Nutr.

[38] Ceesay SM, Prentice AM, Cole TJ, Foord F, Weaver LT, Poskitt EM, Whitehead RG (1997). Effects on birth weight and perinatal mortality of maternal dietary supplements in rural Gambia: 5 year randomised controlled trial. BMJ.

[39] Öztürk HN, Türker PF (2021). Fetal programming: could intrauterin life affect health status in adulthood?. Obstet Gynecol Sci.

[40] Lucas A, Fewtrell MS, Cole TJ (1999). Fetal origins of adult disease-the hypothesis revisited. BMJ.

[41] Martorell R, Ramakrishnan U, Rivera J, Melgar P (1996). Stunting at 3 years of age in Guatemalan girls and birth weight of their children. FASEB J.

[42] Aryastami NK, Shankar A, Kusumawardani N, Besral B, Jahari AB, Achadi E (2017). Low birth weight was the most dominant predictor associated with stunting among children aged 12-23 months in Indonesia. BMC Nutr.

[43] Singh S, Sedgh G, Hussain R (2010). Unintended pregnancy: worldwide levels, trends, and outcomes. Stud Fam Plann.

[44] Brunner Huber LR, Hogue CJ (2005). The association between body weight, unintended pregnancy resulting in a livebirth, and contraception at the time of conception. Matern Child Health J.

[45] Brunner LR, Hogue CJ (2005). The role of body weight in oral contraceptive failure: results from the 1995 national survey of family growth. Ann Epidemiol.

[46] Bajos N, Wellings K, Laborde C, Moreau C (2010). Sexuality and obesity, a gender perspective: results from French national random probability survey of sexual behaviours. BMJ.

[47] Rahman MM (2015). Is unwanted birth associated with child malnutrition in Bangladesh?. Int Perspect Sex Reprod Health.

[48] Singh A, Chalasani S, Koenig MA, Mahapatra B (2012). The consequences of unintended births for maternal and child health in India. Popul Stud (Camb).

[49] (2024). Packaged food: quarterly statement Q4 2021. Euromonitor Passport International London.

[50] Popkin BM (2002). The shift in stages of the nutrition transition in the developing world differs from past experiences!. Public Health Nutr.

[51] Popkin BM, Reardon T (2018). Obesity and the food system transformation in Latin America. Obes Rev.

[52] Monteiro CA, Levy RB, Claro RM, de Castro IR, Cannon G (2011). Increasing consumption of ultra-processed foods and likely impact on human health: evidence from Brazil. Public Health Nutr.

[53] Miller V, Webb P, Micha R, Mozaffarian D (2020). Defining diet quality: a synthesis of dietary quality metrics and their validity for the double burden of malnutrition. Lancet Planet Health.

[54] Inanc N, Aykut M, Cicek B, Şahin H, Yılmaz M, Katrancı D, Tuna R (2005). [Malnutrition status of 0-36 months old children in Kayseri̇ Province and some effecting factors]. Türk Hij Den Biyol Derg.

[55] Castro Martín T (1995). Women's education and fertility: results from 26 Demographic and Health Surveys. Stud Fam Plann.

